# Independent calculation‐based verification of volumetric‐modulated arc therapy–stereotactic body radiotherapy plans for lung cancer

**DOI:** 10.1002/acm2.12900

**Published:** 2020-05-11

**Authors:** Tomohiro Ono, Takamasa Mitsuyoshi, Takashi Shintani, Yusuke Tsuruta, Hiraku Iramina, Hideaki Hirashima, Yuki Miyabe, Mitsuhiro Nakamura, Yukinori Matsuo, Takashi Mizowaki

**Affiliations:** ^1^ Department of Radiation Oncology and Image‐applied Therapy Kyoto University Graduate School of Medicine Kyoto Japan; ^2^ Division of Clinical Radiology Service Kyoto University Hospital Kyoto Japan; ^3^ Division of Medical Physics Department of Information Technology and Medical Engineering Human Health Sciences Kyoto University Graduate School of Medicine Kyoto Japan

**Keywords:** independent verification, lung cancer, secondary treatment planning system, stereotactic body radiotherapy, volumetric‐modulated arc therapy

## Abstract

This study aimed to investigate the feasibility of independent calculation‐based verification of volumetric‐modulated arc therapy (VMAT)–stereotactic body radiotherapy (SBRT) for patients with lung cancer using a secondary treatment planning system (sTPS). In all, 50 patients with lung cancer who underwent VMAT‐SBRT between April 2018 and May 2019 were included in this study. VMAT‐SBRT plans were devised using the Collapsed‐Cone Convolution in RayStation (primary TPS: pTPS). DICOM files were transferred to Eclipse software (sTPS), which utilized the Eclipse software, and the dose distribution was then recalculated using Acuros XB. For the verification of dose distribution in homogeneous phantoms, the differences among pTPS, sTPS, and measurements were evaluated using passing rates of a dose difference of 5% (DD5%) and gamma index of 3%/2 mm (γ3%/2 mm). The ArcCHECK cylindrical diode array was used for measurements. For independent verification of dose‐volume parameters per the patient’s geometry, dose‐volume indices for the planning target volume (PTV) including D_95%_ and the isocenter dose were evaluated. The mean differences (± standard deviations) between the pTPS and sTPS were then calculated. The gamma passing rates of DD5% and γ3%/2 mm criteria were 99.2 ± 2.4% and 98.6 ± 3.2% for pTPS vs. sTPS, 92.9 ± 4.0% and 94.1 ± 3.3% for pTPS vs. measurement, and 93.0 ± 4.4% and 94.3 ± 4.1% for sTPS vs. measurement, respectively. The differences between pTPS and sTPS for the PTVs of D_95%_ and the isocenter dose were −3.1 ± 2.0% and −2.3 ± 1.8%, respectively. Our investigation of VMAT‐SBRT plans for lung cancer revealed that independent calculation‐based verification is a time‐efficient method for patient‐specific quality assurance.

## 1 Introduction

Stereotactic body radiotherapy (SBRT) is an effective treatment for patients with inoperable stage I non‐small‐cell lung cancer (NSCLC).^1^ Previous studies found that SBRT for NSCLC can achieve excellent 3‐year overall survival (OS) and high local control rates with minimal toxicity.^2^, ^3^, ^4^, ^5^ In recent years, volumetric‐modulated arc therapy (VMAT) has been introduced as a form of SBRT for lung cancer treatment^6^ and it is becoming regularly employed owing to its rapid delivery of radiation doses and superior dose conformity.^7^ Since April 2018, VMAT has been used in our institution to treat patients with NSCLC using 6 MV X‐rays delivered by the Vero4DRT linear accelerator system (Mitsubishi Heavy Industries, Ltd., Tokyo, Japan and Brainlab AG, Feldkirchen, Germany).

Patient‐specific quality assurance (QA) should be performed for all radiotherapy plans. For three‐dimensional conformal radiotherapy (3D‐CRT)‐type SBRT for NSCLC, we employed an independent calculation‐based verification using a secondary treatment planning system (sTPS).^8^ The dose difference to the planning target volume (PTV) isocenters delivered by the X‐ray voxel Monte Carlo (XVMC) versus the Acuros XB (AXB) at our institution was found to be −0.3 ± 1.4% (the XVMC and AXB are categorized as type “c” algorithms in which the modeling of secondary electron transport is markedly improved compared to superposition/convolution methods), and we defined the tolerance level as the isocenter dose difference. While measurement‐based patient‐specific QA methods are still widely used for IMRT/VMAT as recommended by the “American Association of Physicists in Medicine” task group (218),^9^ independent calculation‐based verification was one of the patient‐specific QA methods used for the non‐intensity modulated radiotherapy (IMRT) technique.^10^ As such, measurement‐based patient‐specific QA for VMAT‐SBRT is performed at our institution using an ArcCHECK cylindrical diode array (SunNuclear, Melbourne, FL, USA). Although the actual treatment delivery time is shorter owing to VMAT specifications, performing patient‐specific QA consumes more time than does the 3D‐CRT itself.

Recent studies in the medical physics field have explored the efficiency of non‐measurement‐based patient‐specific QA for IMRT‐VMAT.^11^, ^12^ Tachibana et al.^13^ compared secondary checks of 973 treatment planning protocols using computer‐based independent verification for non‐IMRT, IMRT, and VMAT methods, and found that a 5% action level was justifiable for all sites except the lungs. Incidentally, the dose calculation algorithms in their study included the pencil beam method, which is not suitable for lung‐related calculations. Thus, there were large systematic differences in lung site estimates when using computer‐based independent verification because of the large differences in heterogeneity corrections between the primary treatment planning system (pTPS) and verification program. Handsfield et al. found that a new patient‐specific QA procedure for TomoTherapy using log files and secondary Monte Carlo dose calculations was an effective and efficient alternative to the traditional phantom‐based QA method.^14^ However, their analyses required commercially available or special in‐house software. Considering the necessity of patient‐specific QA for IMRT‐VMAT, including for pulmonary and non‐pulmonary sites, we turned our attention to an sTPS for which commercially available software is used in our facility.

This study aimed to investigate the feasibility of using a commercially available software, Eclipse, as an independent calculation‐based verification for lung VMAT‐SBRT. Specifically, dose distributions in a homogeneous phantom and patient geometry were calculated and measured. Such a verification system would be more efficient for evaluating dose distributions in pulmonary sites than computer‐based verification.

## 2 Methods

### 2.A Patient population and data acquisition

In all, 50 consecutive patients with lung cancer who underwent VMAT‐SBRT between April 2018 and May 2019 were included in this study. They comprised 42 men and 8 women with a median age of 81 (range, 58–93) years. Lung tumors in these patients were located in the right upper lobe (14 patients), right middle lobe (eight patients), right lower lobe (six patients), left upper lobe (14 patients), left middle lobe (six patients), and left lower lobe (two patients). This study (R1446) was approved by the institutional review board of the Kyoto University Hospital on January 30, 2018.

With considerations for respiratory‐induced anatomical motion, four‐dimensional (4D) computed tomography (CT) data were acquired using the SOMATOM Definition AS scanner (Siemens, Erlangen, Germany) and the real‐time Positioning Management system (Varian Medical Systems, Palo Alto, CA, USA); the latter illuminated and tracked an infrared reflective marker placed on the patient’s abdomen. The 4D‐CT (slice thickness, 2.0 mm) was performed while the patient breathed freely without audio/visual coaching. CT data were reconstructed in a field of view of 500 mm on a 512 × 512 grid. If the amplitude of respiratory‐induced tumor motion was large, abdominal compression was used to reduce tumor motion.^15^ The range of lung tumor motion, which was evaluated as the mean ± standard deviation (SD) (minimu to–maximum), was 6.0 ± 4.1 (0–20) mm in the superior–inferior direction, 2.5 ± 1.3 (0–6) mm in the left–right direction, and 3.0 ± 1.7 (0–10) mm in the anterior–posterior direction. The maximum and mean intensity projection images were acquired. The dose calculation was performed on the mean intensity projection images.

### 2.B Target delineation and fraction regimens

The internal gross tumor volume (iGTV) was delineated based on the maximum intensity projection as well as the 10 respiratory phase images of the 4D‐CT. The internal target volume was defined by adding a 3 mm margin to the iGTV, while the PTV was created by adding a 5 mm margin to the internal target volume. The prescribed dose was defined as that covering the 95% of the PTV. Of 50 patients, 37 received a prescribed dose of 50 Gy in four fractions, seven received 60 Gy in eight fractions, three received 57.6 Gy in 16 fractions, one received 60 Gy in 10 fractions, one received 65 Gy in 25 fractions, and one received 75 Gy in 25 fractions.

### 2.C Patient‐specific QA

#### 2.C.1 pTPS

Patients who underwent VMAT‐SBRT were treated with the Vero4DRT system; all VMAT‐SBRT plans were created using RayStation version 6.2 (RaySearch Medical Laboratories AB, Stockholm, Sweden), which we considered the pTPS. Collapsed‐Cone Convolution (CCC), version 3.4, was used as the dose calculation algorithm, and the dose calculation grid size was 2.0 mm. All the plans were optimized using a gantry angle sampling of 4° between the control points. VMAT‐SBRT was delivered with 2–6 arcs, including coplanar and non‐coplanar beams.

#### 2.C.2 sTPS

After the VMAT‐SBRT plans were created using the pTPS, all DICOM files (including the CT images, structure files, plan files, and dose files) were transferred from the pTPS to the sTPS, which was Eclipse version 15.6 (Varian Medical Systems, Palo Alto, CA). The dose calculation algorithm was AcurosXB (AXB) version 15.6.05, and the dose calculation grid size was 2.0 mm using dose‐to‐medium with heterogeneity correction. Details of the commissioning of AXB for Vero4DRT including dosimetric evaluation for a heterogeneity phantom were described previously.^8^, ^16^ The dose comparison between pTPS and sTPS was performed in Eclipse.

#### 2.C.3 Measurement‐based patient‐specific QA

For all VMAT‐SBRT plans, measurement‐based patient‐specific QA was performed using ArcCHECK with the acrylic plug. The measured dose distributions were compared with their calculated pTPS and sTPS counterparts using a dose difference (DD) of 3% (DD3%) and 5% (DD5%) as well as gamma indices of 2%/2 mm (γ2%/2 mm), 3%/2 mm (γ3%/2 mm), and 3%/3 mm (γ3%/3 mm). The passing rates for areas receiving isodoses above 10% were calculated using a global difference approach for the absolute dose.

### 2.D Verifications

The following verifications were performed for 50 treatment plans of VMAT‐SBRT:

1. Independent verification based on dose distributions in homogeneous phantoms.
2. Independent verification based on dose‐volume parameters using patient geometry.

#### 2.D.1 Independent verification based on dose distributions in the homogeneous phantom

For this verification, two comparisons were performed. The first was a comparison between measured and calculated dose distributions, including the pTPS and sTPS, as described in “C. Patient‐specific QA” section. Therefore, the dose difference represented the uncertainty in the treatment plan, including the linear accelerator output variations, multileaf collimator position accuracy, or the TPS beam modeling accuracy. The second one was a comparison between the dose distributions for pTPS and sTPS; the dose differences mainly represented the error in the TPS’s model except for the effect of heterogeneity on patient geometry. These differences were evaluated using DD3%, DD5%, γ2%/2 mm, γ3%/2 mm, and γ3%/3 mm.

In addition to the dose index verifications, the similarity of fail points among the pTPS, sTPS, and measurement dose distributions were evaluated by Simpson’s Faunal Resemblance Index (FRI).^17^ The FRI is used to calculate the similarity between pairs of community samples. The FRI takes into account only the number of species occurring in the smaller sample; thus, it is the least influenced by the sample size and emphasizes the similarity of fail points.

The formula used for FRI was as follows:

(1) $$\text{FRI}=\frac{\left|X\cap Y\right|}{min\left(\left|X\right|,\left|Y\right|\right)}$$

where |X ∩ Y| is the number of non‐empty categories in the intersection of distributions X and Y, and min (|X|, |Y|) is the smaller number of the two categories. The FRI ranges from 0 to 1, with a higher value corresponding to a closer similarity. The FRI was calculated for fail points of the DD3% and DD5% dose indices; this evaluation would help predict the uncertainty between the dose of the TPS and that of measurement, without measurement‐based patient‐specific QA.

#### 2.D.2 Independent verification based on dose‐volume parameters according to patient geometry

In general, dose distribution depends on the dose calculation algorithm and patient geometry. To validate the adequacy of the prescription dose, the dose‐volume parameters of pTPS and sTPS were compared, and the dose delivered to the isocenter and dose‐volume parameters were evaluated. For target dose evaluation, the mean dose and the 2% and 95% of the volumes (D_2%_ and D_95%_) of the iGTV and PTV were included and represented by the percentage dose value. For organ dose delivery verification, the present volume irradiated by 20 Gy (V_20Gy_) of the lung (defined as normal lung tissue subtracted from the iGTV), and the maximum dose to the spinal cord were included. The differences in dose‐volume parameters were evaluated by the dosimetric error (DE) and volume error (VE) between the sTPS and pTPS, defined as follows:

(2) $$\text{DE}=\frac{D_{\mathrm{sTPS}}-D_{\mathrm{pTPS}}}{D_{\mathrm{pTPS}}}\times 100$$

(3) $$\text{VE}=\frac{V_{\mathrm{sTPS}}-V_{\mathrm{pTPS}}}{V_{\mathrm{pTPS}}}\times 100$$

where D_sTPS_ and D_pTPS_ are the doses delivered to the sTPS and pTPS targets, respectively, and V_sTPS_ and V_pTPS_ are the volumes of the organs‐at‐risk per the sTPS and pTPS, respectively. Here, the tolerance level of patient‐specific QA was defined as the mean ± SD for the target. Additionally, Student’s *t* test was performed on all 50 treatment plans, and *P* values < 0.05 were considered statistically significant.

The effects of PTV size and the Hounsfield Units (HUs) within the PTV on the DE of the targets were evaluated using the correlations among the DEs of the targets, PTV size, and the mean and minimum HU values in the PTV. The correlation was evaluated by Pearson’s correlation coefficient (CC), the ranges of which were defined as 0.0 < CC <0.4 for weak correlation, 0.4 ≤ CC <0.8 for moderate correlation, and 0.8 ≤ CC <1.0 for strong correlation.

## 3 RESULTS

### 3.A Dose distribution in homogeneous phantoms

**Fig. 1:**
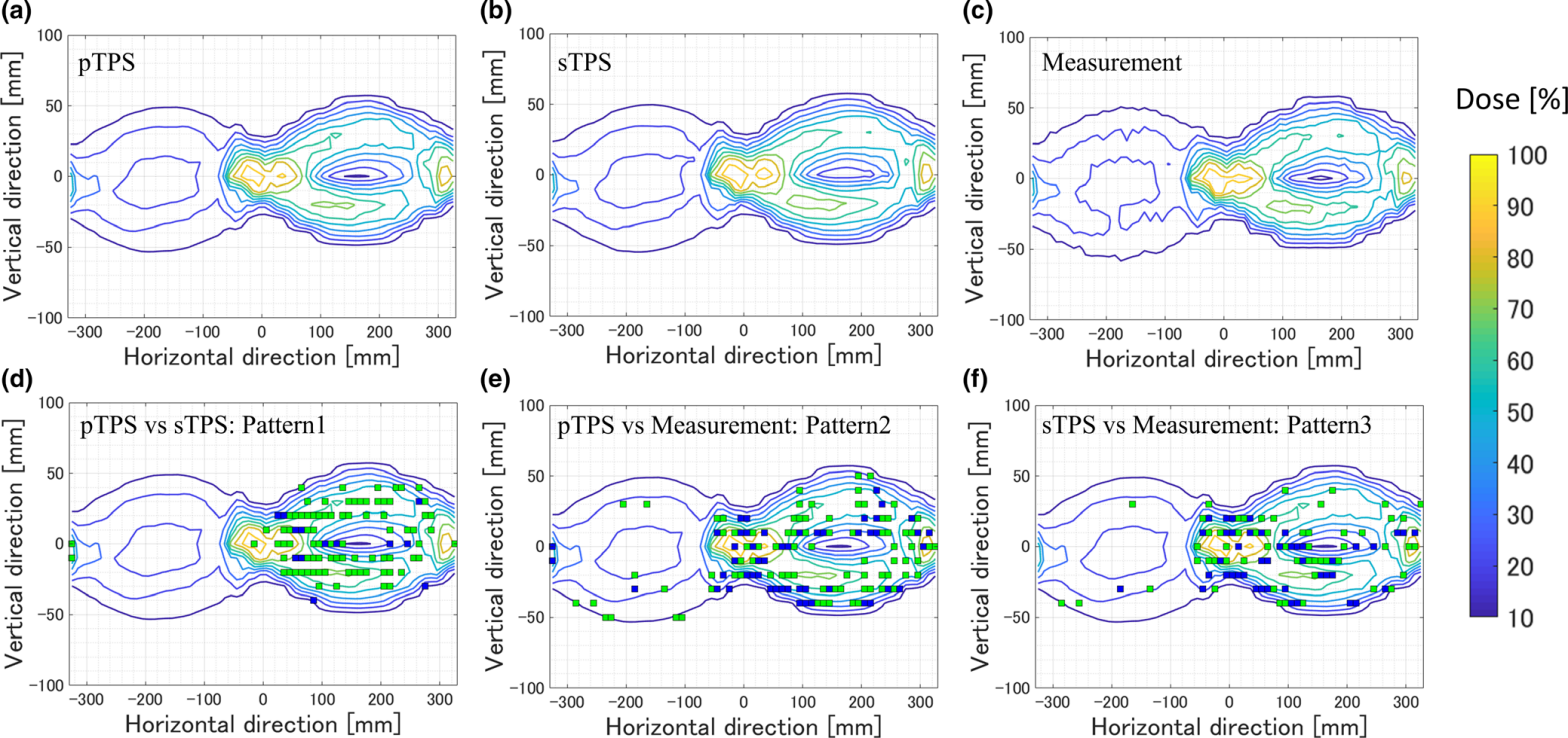
Example of pTPS, sTPS, and actual measurement dose distributions on a homogeneous phantom (a–c). The fail points of the DD3% and DD5% between pTPS and sTPS (defined as pattern 1), pTPS and measurement (defined as pattern 2), and sTPS and measurement (defined as pattern 3) are shown in panels (d), (e), and (f), respectively. Abbreviations: pTPS, primary treatment planning system; sTPS, secondary treatment planning system; DD, dose difference.

Figure 1(a)–1(c) shows an example of the patterns for pTPS, sTPS, and the actual measurement results in a homogeneous phantom. The dose distributions were normalized at the maximum dose in each case and displayed as beam eye view distributions throughout the entire arc delivery using ArcCHECK. Figure 1(d)–1(f) shows the fail points of DD3% and DD5% between the pTPS and sTPS (pattern 1), pTPS and measurement (pattern 2), and sTPS and measurement (pattern 3). Table 1 shows the dose indices for the dose distributions per pTPS, sTPS, and the actual measurement for 50 patients. The mean dose index derived from pattern 3 was slightly better than that derived from pattern 2. On the other hand, the mean dose index of pattern 1 was higher than those of patterns 2 and 3. The uncertainty of the pattern 1 treatment plan was larger than the error in the TPS’s calculation.

**Table I:** Dose indices for pTPS, sTPS, and measurement dose distributions for 50 treatment plans.

	DD3%			DD5%			γ2%/2 mm			γ3%/2 mm			γ3%/3 mm		
	Mean ± SD (%)	max (%)	min (%)	Mean ± SD (%)	max (%)	min (%)	Mean ± SD (%)	max (%)	min (%)	Mean ± SD (%)	max (%)	min (%)	Mean ± SD (%)	max (%)	min (%)
pTPS vs sTPS (pattern 1)	96.3 ± 5.4	100.0	71.0	99.2 ± 2.4	100.0	85.1	96.2 ± 5.1	100.0	72.3	98.6 ± 3.2	100.0	80.2	99.2 ± 2.0	100.0	87.5
pTPS vs measurement (pattern 2)	81.2 ± 6.5	96.5	65.7	92.9 ± 4.0	99.6	79.0	88.4 ± 4.8	96.7	79.7	94.1 ± 3.3	99.5	86.5	98.1 ± 1.5	100.0	92.8
sTPS vs measurement (pattern 3)	82.2 ± 7.2	95.0	63.3	93.0 ± 4.4	100.0	82.2	90.1 ± 5.2	96.6	69.4	94.3 ± 4.1	99.1	77.8	98.2 ± 2.0	100.0	87.8

Abbreviations: pTPS, primary Treatment Planning System; sTPS, secondary Treatment Planning System; DD, dose difference; γ, gamma index; SD, standard deviation.

**Fig. 2:**
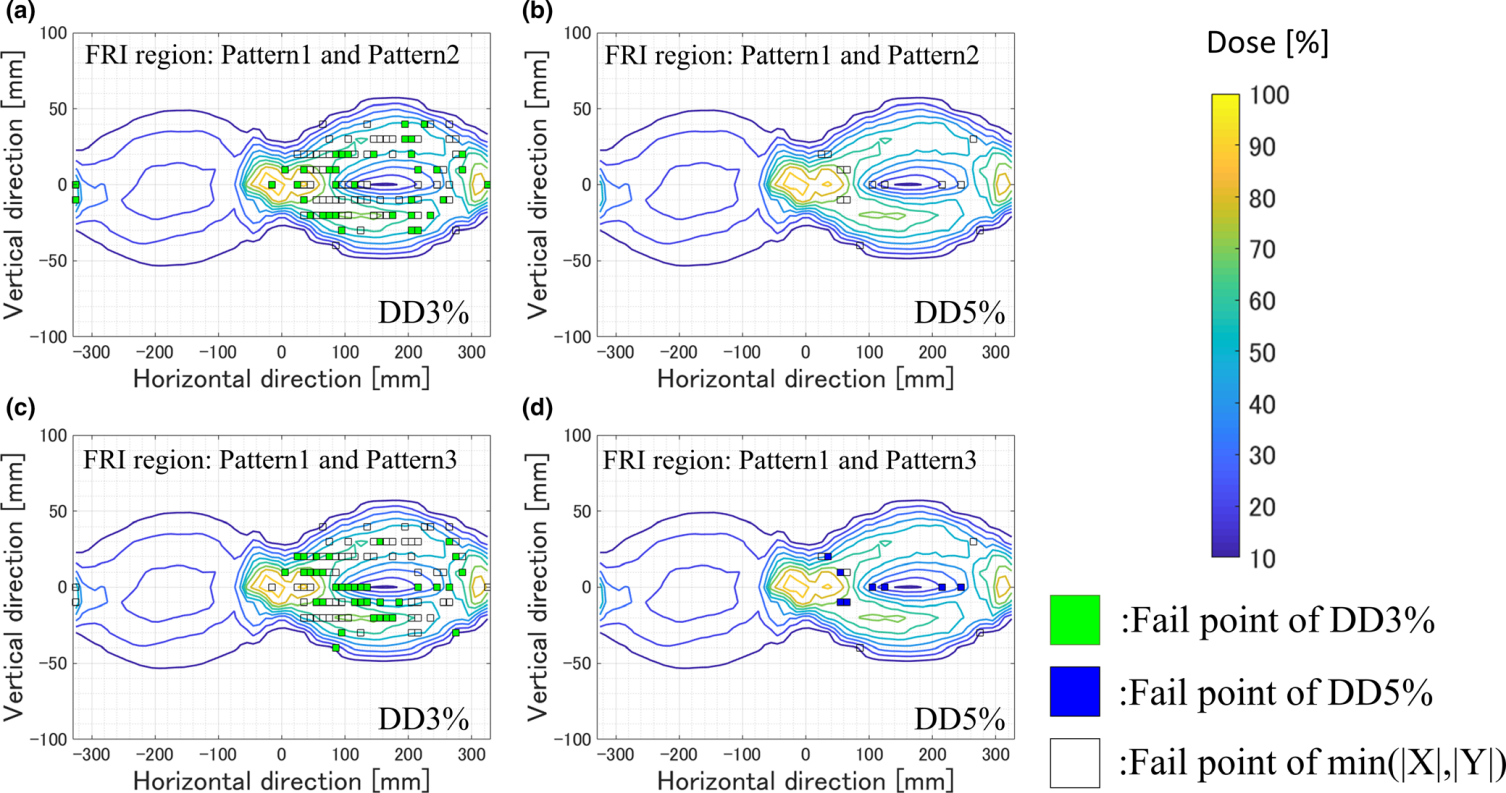
Example of the FRI regions between patterns 1 and 2 for DD3% (a) and DD5% (b) as well as between patterns 1 and 3 for DD3% (c) and DD5% (d). Pattern 1, 2, and 3 were defined between pTPS and sTPS, pTPS and measurement, and sTPS and measurement, respectively.

Figure 2 shows the examples of FRI region comparisons between patterns 1 and 2 and between patterns 1 and 3 in terms of the DD3% and DD5%. In this example, the min (|X|, |Y|) represented the total fail points in pattern 1 because this number was smaller than that of the total fail points in patterns 2 or 3. In the FRI region of DD3%, the same fail points were observed in patterns 1 and 2 and for patterns 1 and 3. In the FRI region of DD5%, patterns 1 and 3 shared a common region although patterns 1 and 2 did not. Table II shows the FRIs between patterns 1 and 2 as well as those between patterns 1 and 3 in terms of the DD3% and DD5%. The FRIs were calculated by excluding the 100% passing rates for DD3% and DD5% (as these contained no fail points). For both DD3% and DD5%, the FRIs between patterns 1 and 3 were higher than those between patterns 1 and 2, especially for the DD5%.

**Table II:** FRIs between patterns 1 and 2 and between patterns 1 and 3 in terms of the DD3% and DD5% for 50 treatment plans.

	FRI					
	DD3%			DD5%		
	Mean ± SD	max	min	Mean ± SD	max	min
pTPS vs sTPS: Pattern 1 and pTPS vs measurement: Pattern2	0.39 ± 0.25	1.00	0.00	0.05 ± 0.10	0.50	0.00
pTPS vs sTPS: Pattern 1 and sTPS vs measurement: Pattern 3	0.44 ± 0.29	1.00	0.00	0.48 ± 0.41	1.00	0.00

Abbreviations: FRI, Faunal Resemblance Index; pTPS, primary treatment planning system; sTPS, secondary treatment planning system; DD, dose difference; γ, gamma index; SD, standard deviation.

### 3.B Dose‐volume parameters in patient geometry

**Fig. 3:**
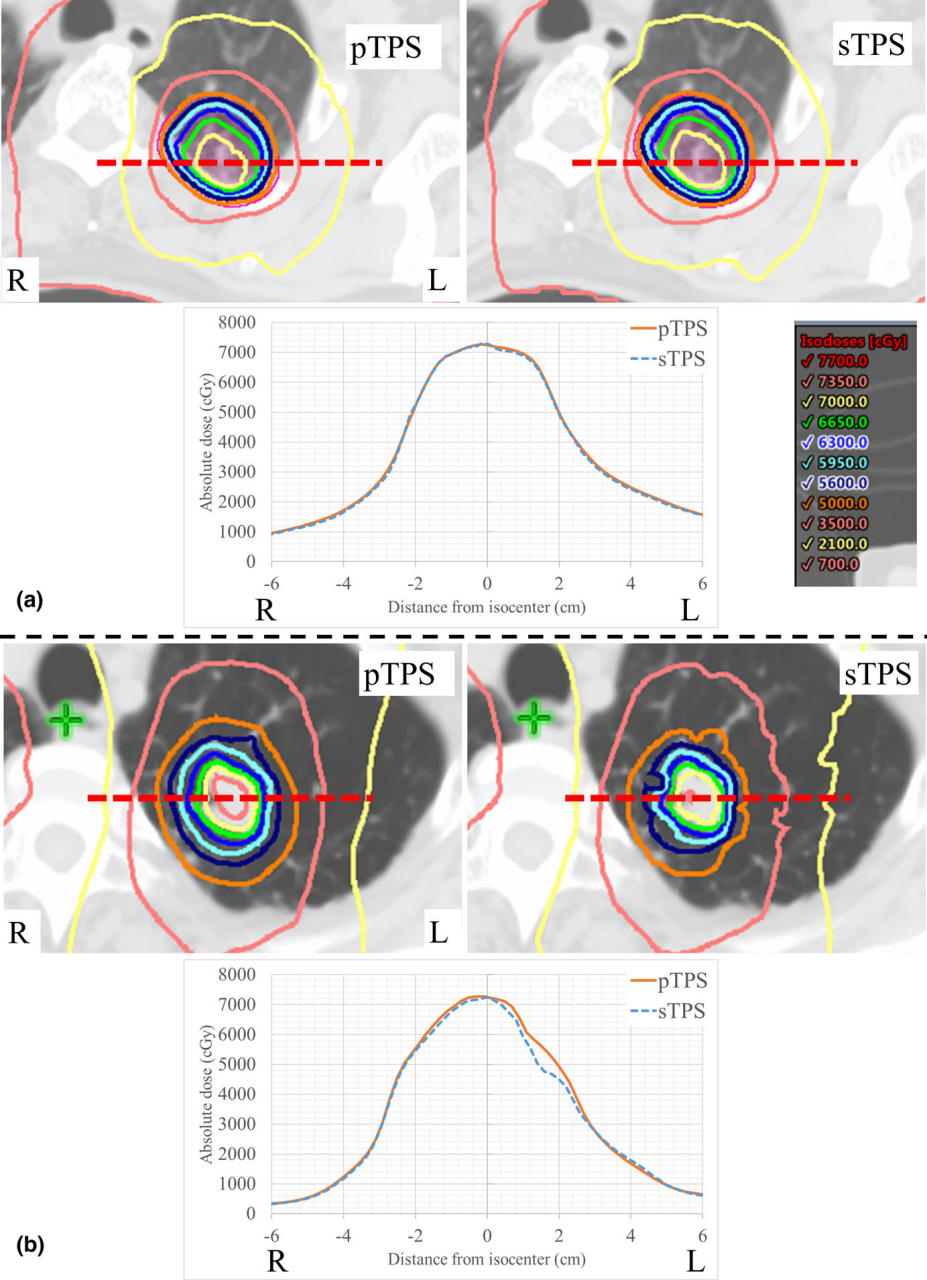
Examples of pTPS and sTPS dose distributions and dose profiles according to patient geometry. In (a), the mean and minimum CT values were −205.9 and −917.0 HU, respectively, and the dose profiles were comparable. In (b), the mean and minimum CT values were −694.3 and −1000.0 HUs, respectively, and the dose profiles were not comparable. Abbreviations: HU, Hounsfield units; pTPS, primary treatment planning system; sTPS, secondary treatment planning system

Table III summarizes the PTV size categories and average HU values within PTV for the 50 patients. The PTVs, as well as the average HU values, were 45.2 ± 36.0 cm^3^ (range, 5.9–162.9 cm^3^) and − 541.3 ± 193.3 HU (range, −852.9 to −125.6 HU). Figure 3 shows the examples of dose distributions and profiles of the pTPS and sTPS in the same patient geometry. In [Fig. 3(a)], the lung tumor was located in the left upper lobe, and the PTV, mean CT, and minimum CT values were 55.1 cm^3^, −205.9 HU, and − 917.0 HU, respectively. Dose profiles were similar between the pTPS and sTPS. In contrast, the dose profiles were not similar between pTPS and sTPS as shown in [Fig. 3(b)]; in this patient, the lung tumor was located in the left upper lobe, and the PTV, mean CT, and minimum CT values were 40.5 cm^3^, −694.3 HU, and −1000.0 HU, respectively. The DE and VE of dose‐volume parameters of the isocenter, iGTV, PTV, and lung and spinal cord are listed in Table 4. Doses to the isocenter and target volume per the sTPS were significantly smaller than those per the pTPS. On the other hand, the dose to the lung per the sTPS was larger than that per the pTPS; this difference was significant except for the V_20Gy_ of the lung. Table 5 shows the CCs among the DE of the targets, PTV size, and the average HU values in the PTV. For the DE of the isocenter dose, the CC of the PTV size (e.g., 0.36) was higher than that of the CT values (e.g., mean: 0.09 and SD: 0.16). As for the DE of the target, most CCs had higher values than those of the PTV size. For the prescribed dose index of the PTV D_95%_, a CC of 0.50 was the highest for the mean clinical target volume.

**Table III:** Lesions categorized by PTV size and average HU value within PTV.

PTV size (cm^3^)	Number of lesions
≤20	11
20<, ≤35	14
35<, ≤50	9
50<, ≤75	6
75<	10
Average HU value within PTV (HU)	Number of lesions
≤−600	25
−600 <, ≤−500	5
−500 <, ≤−400	6
−400 <, ≤−300	5
−300 <	9

Abbreviations: PTV, planning target volume.

**Table IV:** DE and VE of dose‐volume parameters of the isocenter, iGTV, PTV, and the lung and spinal cord for 50 treatment plans.

			mean	SD	max	min	*P* value
DE (%)	isocenter dose (%)		−2.3	1.8	0.6	−8.6	<0.05
	iGTV	D_99%_ (%)	−2.9	3.2	12.4	−11.2	<0.05
		D_95%_ (%)	−3.0	2.0	0.4	−8.5	<0.05
		D_mean_ (%)	−2.5	1.6	1.3	−7.9	<0.05
		D_2%_ (%)	−2.0	1.8	2.0	−8.5	<0.05
		D_max_ (%)	−1.5	2.1	1.7	−8.2	<0.05
	PTV	D_99%_ (%)	−3.1	2.5	−1.0	−16.5	<0.05
		D_95%_ (%)	−3.1	2.0	−0.2	−11.5	<0.05
		D_mean_ (%)	−3.0	2.0	0.6	−10.8	<0.05
		D_2%_ (%)	−2.1	1.6	2.4	−7.7	<0.05
		D_max_ (%)	−1.5	2.0	1.7	−8.2	<0.05
VE (%)	Lung	V_20Gy_ (%)	0.3	7.3	0.2	−0.2	0.96
		V_10Gy_ (%)	1.6	3.1	10.8	−7.6	<0.05
		V_5Gy_ (%)	2.5	2.1	9.3	−4.0	<0.05
		D_mean_ (%)	5.1	5.4	17.7	−13.8	<0.05
	Spinal cord	D_max_ (%)	−0.7	2.5	4.5	−9.0	<0.05

Abbreviations: DE, dosimetric error; VE, volumetric error; iGTV, internal gross tumor volume; PTV, planning target volume; SD, standard deviation.

**Table V:** CCs among the DEs of the targets, PTV, and average HU values in PTV for 50 treatment plans.

			CCs		
			PTV size (cm^3^)	Average HU value within PTV (HU)	
				average	SD
DE (%)	isocenter dose (%)		0.36	0.09	0.16
	iGTV	D99 (%)	0.40	0.57	0.52
		D95 (%)	0.38	0.56	0.33
		mean (%)	0.35	0.49	0.32
		D2 (%)	0.26	0.27	0.32
		max (%)	0.29	0.25	0.26
	PTV	D99 (%)	0.26	0.42	0.43
		D95 (%)	0.38	0.50	0.40
		mean (%)	0.38	0.39	0.40
		D2 (%)	0.26	0.33	0.27
		max (%)	0.29	0.23	0.27

Abbreviations: CC, correlation coefficient; DE, dosimetric error; iGTV, internal gross tumor volume; PTV, planning target volume; SD, standard deviation.

## 4 Discussion

We performed independent calculation‐based verifications of VMAT‐SBRT plans for lung cancer. The benefit of this approach is that it allows to evaluate VMAT‐SBRT plan quality without actual dosimetric measurements. Moreover, we confirmed that the DE of the target dose‐volume parameters did not deviate significantly, and that independent calculation‐based verification was useful for evaluating dose distributions in pulmonary sites.

In general, dose distributions are evaluated with homogeneous phantoms when performing patient‐specific QA of IMRT or VMAT. Anjum et al. reported independent calculation‐based verification of IMRT for 24 patients using homogeneous phantoms.^18^ They used the Nomos Corvus (Corvus 6.2, Nomos Corp., Cranberry Township, PA) and Eclipse version 8.1.17 as their pTPS and sTPS, respectively; however, this study reported no details regarding the calculation algorithm. They concluded that the Eclipse‐based sTPS was an accurate, robust, and time‐efficient method for patient‐specific IMRT QA. In our study, we also found that the dose indices obtained using AXB were better than those of the CCC when comparing each of these to the actual measurement. Moreover, our study is the first one to evaluate the fail points among dose distributions of pTPS, sTPS, and actual measurement. In general, the sTPS using the AXB had good agreement with the measured dose distribution; this agreement was better than that of the pTPS using the CCC.^19^ Thus, the fail points derived from the sTPS vs. actual measurement ought to reflect the uncertainty of the treatment plan more reliably than those derived from the pTPS vs. measurement. We also found that the FRI for the DD5% for patterns 1 vs. 3 was 0.48 ± 0.41. In other words, most of the fail points in relative to DD5% are related to the uncertainty in the treatment planning dose calculation algorithms. Therefore, when considering calculation‐based verification without actual measurements, evaluating the gamma passing rate between pTPS and sTPS is useful to determine treatment plan uncertainty. In addition, we found that the FRI between patterns 1 and 2 as well as those between patterns 1 and 3 were lower than 1. This was because the calculation and measurement uncertainties had different factors, for example, beam modeling accuracy or measurement device inaccuracies. Un‐passing points of FRI means different cause of fail point in dose differences. In other words, the common fail points mean high risk of different points between calculation and measurement uncertainties. The use of FRI would contribute to the better judgment of pure plan uncertainty.

Several studies investigated the dose‐volume parameters in terms of patient geometry. Mampuya et al. investigated the D_95%_ dose difference for the PTV between the analytical anisotropic algorithms (AAA) and AXB using conventional SBRT plans for 37 patients with lung cancer.^20^ The dose differences were −0.3 ± 1.4% for the isocenter and −1.3 ± 1.8% for the D_95%_ of the PTV. Tsuruta et al. performed independent calculation‐based verification of conventional SBRT plans for lung cancer,^8^ and concluded that the dose to the isocenter as well as the dosimetric parameters of D_50%_ and D_95%_ were useful for independent verification. In our study presented here, we also found that the dose differences and deviations were the smallest at the isocenter.

The dose differences among dose calculation algorithms are large for non‐homogeneous regions such as the lung. Tsuruta et al. reported significant differences between dose calculation algorithms around the PTV in low‐density regions when performing dosimetric comparisons of the AAA, AXB, and XVMC for lung cancer.^16^ In particular, the dose difference between the AAA and XVMC outside the PTV was up to 15.5%. As shown in [Fig. 3(b)], we also found a dose difference within the low‐density region. In terms of applying the DE or VE of the dose‐volume parameter to the patient‐specific QA tolerance level, small deviations in the DE or VE as well as excluding uncertainties between the pTPS and sTPS appear to be acceptable. Thus, we concluded that the tolerance level should be defined as the DE of the isocenter rather than that of the outside of the PTV (such as the D_95%_).

Considering that the time consumption of the experimental method for the patient‐specific QA is increasing in the frequency of IMRT or VMAT use in the clinic, the patient‐specific QA method should be a more efficient QA procedure. As the efficient QA procedure, we previously developed a patient‐specific QA prediction method incorporating the gamma passing rate in 600 VMAT plans.^21^ The DD5% and γ3%/3 mm were predicted using plan complexity parameters via a neural network, and their prediction errors were −0.2 ± 2.7% and −0.2 ± 2.1%, respectively. This QA prediction method may contribute to simpler and more efficient patient‐specific QA strategies without requiring actual dosimetric measurements.

We acknowledge certain limitations in our study. Independent calculation‐based verification cannot replace measurements done with IMRT or VMAT equipment and it cannot confirm that the correct multileaf collimator position, gantry, and collimator parameters have been transferred to the treatment console. The lack of data transfer verification would cause significant mishaps for patients, as was previously reported by the New York Times.^22^ However, such errors can be detected using software‐based methods.^23^ When applying independent calculation‐based verification for IMRT or VMAT, rigorous QA program such as machine QA and data transfer QA should be established for implementation at the pre‐treatment stage.

## 5 CONCLUSIONS

The findings of this study were as follows: (a) calculating the difference in the gamma passing rates of pTPS and sTPS is useful for determining treatment plan uncertainty, (b) small deviations in the DEs of target dose‐volume parameters in pulmonary sites are acceptable, and (c) isocenter dose verification is suitable for defining the tolerance level for patient‐specific QA. Independent calculation‐based verification can be used as a time‐efficient method for patient‐specific QA under the condition that pre‐treatment verification is performed to confirm the data transfer.

## Conflict of interest

The authors have no relevant conflicts of interest to disclose.
